# Prevalence and antibiotic resistance of *Pseudomonas aeruginosa* in water samples in central Italy and molecular characterization of *opr*D in imipenem resistant isolates

**DOI:** 10.1371/journal.pone.0189172

**Published:** 2017-12-06

**Authors:** Giuditta Fiorella Schiavano, Elisa Carloni, Francesca Andreoni, Silvia Magi, Maria Chironna, Giorgio Brandi, Giulia Amagliani

**Affiliations:** 1 Department of Biomolecular Sciences, Toxicological, Hygienistic and Environmental Sciences Unit, University of Urbino Carlo Bo, Urbino, PU, Italy; 2 Department of Biomolecular Sciences, Section of Biotechnology, University of Urbino Carlo Bo, Fano, PU, Italy; 3 Dipartimento provinciale ARPAM di Pesaro, Servizio Acque, Pesaro, Italy; 4 Department of Biomedical Science and Human Oncology-Hygiene Section, Aldo Moro University of Bari, Bari, Italy; Seconda Universita degli Studi di Napoli, ITALY

## Abstract

**Scope:**

This study aimed to analyse the prevalence, antibiotic resistance and genetic relatedness of *P*. *aeruginosa* isolates obtained from potable and recreational water samples (n. 8,351) collected from different settings (swimming pools, n. 207; healthcare facilities, n 1,684; accommodation facilities, n. 1,518; municipal waterworks, n. 4,500; residential buildings, n. 235). Possible mechanisms underlying resistance to imipenem, with particular focus on those involving *opr*D-based uptake, were also explored.

**Methods and results:**

Isolation and identification of *Pseudomonas aeruginosa* was performed according to the standardized procedure UNI EN ISO 16266:2008 followed by PCR confirmation. Antibiotic Susceptibility testing was conducted according to EUCAST standardized disk diffusion method. Genetic relatedness of strains was carried out by RAPD. The sequence of the *opr*D gene was analyzed by standard method. Fifty-three samples (0.63%) were positive for *P*. *aeruginosa*, of which 10/207 (4.83%) were from swimming pools. Five isolates (9.43%) were resistant to imipenem, one to Ticarcillin + Clavulanate, one to both Piperacillin and Ticarcillin + Clavulanate. The highest isolation rate of imipenem resistant *P*. *aeruginosa* was observed in swimming pool water. Identical RAPD profiles were found in isolates from the same location in the same year or even in different years.

**Conclusions:**

Imipenem resistant strains were identified as carbapenemase-negative and resistance has been associated with inactivating mutations within the *opr*D gene, with a concomitant loss of porin. RAPD results proved that a water system can remain colonized by one strain for long periods and the contamination may be difficult to eradicate.

This study has revealed the presence of *P*. *aeruginosa* in different water samples, including resistant strains, especially in swimming pools, and confirmed the role of porins as a contributing factor in carbapenem resistance in Gram-negative bacteria.

## Introduction

*Pseudomonas aeruginosa* is an opportunistic human pathogen implicated in a variety of acute and chronic infections such as respiratory, urinary tract and gastrointestinal infections as well as bacteremia. It is mainly found in subjects with compromised host defenses, e.g., cancer, HIV and cystic fibrosis (CF) patients [1], and is a significant cause of morbidity and mortality.

It is widespread in the environment, particularly in a variety of water sources such as hospital [2] and municipal drinking water systems [3], healthcare facilities [2,4], accommodation facilities [5], as well as in swimming pools and hot tubs [6,7], where *P*. *aeruginosa* is also a major cause of skin infections such as folliculitis and external otitis [8,9]. In these water systems, *P*. *aeruginosa* has the ability to grow [10–12] and form biofilms [13].

In Italy the quality of water intended for human consumption is ensured by Legislative Decree n.31/2001 [14] and its subsequent amendments, Legislative Decree n.27/2002 [15], while the microbiological criteria for swimming pool water are established by Italian Guidelines [16].

For *P*. *aeruginosa* in swimming pools, there is a maximum permissible limit of zero per 100 ml of inlet water and ≤1 cfu (colony-forming units) per 100 ml of pool water.

Because of its ubiquity, enormous versatility, intrinsic tolerance to many detergents, disinfectants and antimicrobial compounds, *P*. *aeruginosa* is difficult to control. Infections caused by this pathogen are often difficult to treat because of the bacterium’s drug and multidrug-resistant (MDR) phenotypes [17]. Indeed, in the last few years *P*. *aeruginosa* has shown increasing resistance to many antimicrobials, included carbapenems, a class of β-lactam antibiotics widely used in clinical settings [18]. Moreover, a recent study reported that 96% of *P*. *aeruginosa* isolates from swimming pools and hot tubs were found to be multidrug resistant [19], including resistance to front-line antipseudomonal drugs, with the highest percentage of isolates proving resistant to imipenem, β-lactam antibiotic of the carbapenem class.

In 2015, 30 European Union/ European Economic Area (EU/EEA) countries reported 12,689 *P*. *aeruginosa* isolates with antimicrobial susceptibility testing (AST) information for carbapenems (imipenem or meropenem). The number of carbapenem resistant isolates reported per country ranged from 12 to 1,925.

The national percentages of carbapenem-resistant *P*. *aeruginosa* isolates ranged from 0% (Iceland) to 66.3% (Romania), with a 23% prevalence in Italy [20].

Imipenem resistance can involve low permeability, the activity of an inducible β-lactamase [21], and multidrug efflux systems, but the most common mechanism underlying resistance involves the loss of OprD porins from the outer membrane [22], which can occur at the transcriptional or translational level or through the emergence of mutations in the *opr*D gene [23].

The aim of this study was to investigate the prevalence, antimicrobial susceptibility profiles and genetic relatedness of *P*. *aeruginosa* isolates obtained from water samples collected from different locations in the Marches Region, Central Italy.

Possible mechanisms underlying resistance to imipenem, with particular focus on those involving *opr*D-based uptake, were also explored.

## Materials and methods

### Collection of water samples

A total of 8,351 water samples were collected in a wide range of different settings in the Marches Region between 2012–2015: 207 samples in 41 public swimming pools; 1,684 samples in 7 healthcare facilities (hospitals, residential care homes); 1,518 samples in 72 accommodation facilities (campgrounds, hotels, residences, temporary guesthouses); 4,500 samples in 2 municipal waterworks; 235 samples in 30 residential buildings (households).

These samples were collected from Ancona, Ascoli Piceno, Fermo, Macerata, Pesaro-Urbino districts of Marches Region, except for swimming pool and municipal waterworks, which were from Pesaro-Urbino. This is a territorial area of about 9 365.86 km^2^ of Central-Eastern Italy. In the study facilities, water samples were taken from taps, showers and drinking fountains and collected in sterile bottles after 1–5 min of free flow, after disinfection of sample port by 70% ethanol (plastic tubes) or by flaming (metal tubes). For water samples a solution of 0.1 N of sodium thiosulphate was added to neutralize any residual chlorine. Samples were transported into the laboratory using a portable cooler (4–6°C) and analysed immediately.

The data reported in this study have been obtained through the official activity of the Azienda Sanitaria Unica Regionale (ASUR) and the Agenzia Regionale per la Protezione Ambientale delle Marche (ARPAM), which are the authorities responsible for water sampling and analysis. Further specific permissions are not required.

A complete list of sampling locations including isolated strains and facility identification is provided in S1 Table.

### Isolation and identification of *Pseudomonas aeruginosa*

Isolation and identification of *Pseudomonas aeruginosa* was performed according to the standardized procedure UNI EN ISO 16266:2008 [24]. Briefly, 100 mL of each water sample was filtered with a cellulose ester membrane (0.45 μm porosity, 47 mm diameter; Millipore, Billerica, MA, USA), which was then placed onto a Pseudomonas Agar with Pseudomonas CN Supplement (PACN) (Oxoid, Basingstoke, UK) plate.

PACN plates were incubated at 35 ± 1°C for 44 ± 4 h before the counting of colonies. Blue/green pyocyanin-producing colonies were counted as confirmed *P*. *aeruginosa* according to UNI EN ISO 16266:2008 [24]. Fluorescent non-pyocyanin-producing or reddish brown colonies were recorded as presumptive *P*. *aeruginosa* and subjected to confirmation tests according to UNI EN ISO 16266:2008 [24].

### Real-time PCR confirmation of *Pseudomonas aeruginosa* isolates

All stocked isolates were tested for species confirmation by amplification of a specific fragment of the *ecf*X gene. DNA was obtained from each isolate, subcultured on Tryptone Soya Agar (TSA, Thermo Scientific—Oxoid, Basingstoke, UK), by colony lysis by boiling. Lysates were then amplified with primers ecfXF-ecfXR (final concentration 0.4 μM each) and dual labeled probe ecfX-TM (0.16 μM), according to Amagliani et al. [25], with the Hot-Rescue Real-Time PCR Kit (Diatheva, Fano, Italy). Amplification reactions were conducted in a Rotor Gene 3000A (Corbett Research, Sydney, Australia) with the following thermal protocol: denaturation at 95°C for 10 min; 40 cycles at 95°C for 15 s and 60°C for 1 min. Negative (H_2_O) and positive (*P*. *aeruginosa* ATCC 10145 DNA) controls were used in each amplification run and analyzed in the Green channel along with the samples.

### RAPD fingerprinting of *Pseudomonas aeruginosa* isolates

*P*. *aeruginosa* isolates were grown in 5 ml Pseudomonas Selective Broth (Biolife, Milan, Italy) with incubation at 35°C overnight. DNA was then extracted from bacterial pellets using the Bacterial Genomic DNA Isolation Kit (Norgen Biotek, Thorold, Canada) and its concentration estimated through the NanoDrop ND-1000 System (NanoDrop Technologies, Wilmington, DE). RAPD (Random Amplification of Polymorphic DNA) typing was carried out according to Lanotte et al. [26] with 272 primer (5’-AGCGGGCCAA-3’) and 40 ng DNA of each strain. PCR products (12 μl) were run on 1.5% agarose gel with GeneRuler 100 bp DNA Ladder (Thermo Scientific) and analyzed on a Gel Doc 2000 apparatus using Quantity One Quantitation Software (Bio-Rad, Hercules, CA, USA). The molecular size (bp) of each potential band position was determined across all RAPD-PCR profiles. At each band position, two possible alleles were considered either present (a score of 1) or absent (a score of 0). Different RAPD profiles were designated by different scores and classified as different genotypes.

A dendrogram was constructed through the MVSP software (ver. 3.22) (available at http://mvsp.software.informer.com/3.2/). After construction of a similarity matrix, data were cluster analyzed using the UPGMA (Unweighted Pair Group Method with Arithmetic Mean) method with similarity of distance according Jaccard’s coefficient.

### Antibiotic Susceptibility testing (AST)

All *P*. *aeruginosa* isolates (n. 53) were tested for antibiotic sensitivity using the European Committee on Antimicrobial Susceptibility Testing (EUCAST) standardized disk diffusion method [27]. These tests were performed on Mueller-Hinton agar (Difco, Michigan, USA) using the disk diffusion technique by Kirby-Bauer. From the exponential bacterial growth (18–24 hours) in non-selective agar plates, colonies were suspended in 5 ml sterile saline (0.85% NaCl), adjusted to 0.5 McFarland scale turbidity (∼10^8^CFU/ml) and then seeded in Mueller-Hinton agar with the aid of a sterile swab. Within 15 min, antimicrobial disks containing antibiotic were applied to the surface of the agar plates. All plates were incubated at 35°C ± 1° for 16–18 hours. Zones of inhibition around the disk were measured and interpreted as proposed by the EUCAST breakpoint criteria (http://www.eucast.org.).

Sixteen antimicrobial molecules, belonging to 6 different classes, were tested: Penicillins: Piperacillin 30 μg, Piperacillin + Tazobactam 30 + 6 μg, Ticarcillin + Clavulanate 75 + 10 μg, Ticarcillina 75 μg; Cephalosporins: Cefepime 30 μg, Ceftazidime 10 μg; Carbapenems: Doripenem 10 μg, Imipenem 10 μg, Meropenem 10 μg; Monobactams: Aztreonam 30 μg; Fluoroquinolones: Ciprofloxacin 5 μg, Levofloxacin 5 μg; Aminoglycosides: Amikacin 30 μg Gentamicin 10 μg Tobramycin 10 μg, Netilmicin 10 μg (Biolife, Milan, Italy)

The reference strain of *P*. *aeruginosa* ATCC 27853 was used as a quality control in all experiments. A control plate without antibiotics was included in each series.

Each experiment was performed three times.

### PCR detection of β-lactamases genes

The Xpert Carba R assay (Cepheid, Sunnyvale, CA, USA), which detects and differentiates the most prevalent gene families associated with *P*. *aeruginosa* resistance to carbapenems (*bla*_KPC_, *bla*_NDM_, *bla*_IMP_, *bla*_OXA-48-like_, and *bla*_VIM_), was used on isolates showing resistance to carbapenems in disk diffusion tests according to the manufacturer’s instructions.

### DNA sequencing and analyses of sequence data

The sequence of the *opr*D gene was analyzed in all isolates from swimming pools and one resistant strain from healthcare facilities. The entire coding sequence and its flanking regions, including the promoter, were amplified with specific primers: OprD_PCR F (5’-CGTCGCTTCGGAACCTCAACTA-3’) and OprD_PCR R (5’- GCCGTGACCTCGAACCTGA-3’). The PCR was performed in a final volume of 25 μl, using 200 nM of each primer and 2.5 U HotStarTaq DNA Polymerase (Qiagen), under the following conditions: 95°C for 15 min, 35 cycles at 94°C for 1 min, 63°C for 30 s and 72°C for 3 min. The PCR products were separated on 1% agarose gel, purified using MinElute Gel Extraction Kit (Qiagen), and directly sequenced. Specific primers for the target *opr*D sequence were designed and used for the DNA sequencing analyses (Table 1). Primers targeting the insertion sequences (IS) within the *opr*D gene were also designed and used in strains indicated in Table 1. Sequencing was performed with the BigDye Terminator Cycle Sequencing Kit v1.1 and run in the ABI PRISM 310 Genetic Analyzer (Applied Biosystems).

**Table 1:** Primers used for sequencing.

Name	5’-3’	Strains^a^
**Specific primers for the target *oprD* gene**		
oprD_PCR F	CGTCGCTTCGGAACCTCAACTA	All strains
oprD_PCR R	GCCGTGACCTCGAACCTGA	All strains
oprD_seq F	CCTGAAGCTCGACGGCACCTC	All strains
oprD_seq R	TCGCCGTAGCCGTAGTTCTTA	All strains
oprD_seq F2	CAGCCGCCTGTTCCCGCAGACC	All strains
oprD_seq R2	GGTAGGCCAAGGTGAAAGTGTG	All strains
oprD_seq F3	GCTGCTCCGCAACTACTATTTC	All strains
**Specific primers for the insertion sequence**		
oprD_seq R4	GGCGTGCCGTCGTTCATCACTG	16/PN/12, 53/PN/13, 482/PA/15
oprD_seq F5	AGAAGTTGGTCGAGCGTGCG	482/PA/15
oprD_seq R5	TGCTTGCCTTCGGTGAAGTG	482/PA/15
oprD_seq F6	CCCGCTCGATACGGTGTATCC	482/PA/15
oprD_seq R6	GGCGTTGGTGGTGTAGATCACC	482/PA/15
oprD_seq F7	TTGATCACCAACTCAGCATCCG	16/PN/12, 53/PN/13
oprD_seq R7	TGATGTCGGACGAGTTCAATCG	16/PN/12, 53/PN/13
oprD_seq R8	CAACGTCGTTCCTTTACGCCCG	16/PN/12

^**a**^Isolates used for sequence analysis of *opr*D gene: 4/PN/12, 10/PN/12, 13/PN/12, 16/PN/12, 25/PN/12, 33/PN/12, 37/PN/13, 53/PN/13, 10/PN/14, 7/PN/15, and 482/PA/15.

### Computer analyses of sequence data

The obtained nucleotide sequences were analyzed by BioEdit Sequence Alignment Editor version 7.2.5 software. Comparison of nucleotide sequences against sequence database was performed with BLAST. The translation of the nucleic acids sequences into amino acids was performed using ExPASy Bioinformatics Resource Portal (http://web.expasy.org/translate). The resulting protein sequences were then aligned and analyzed using Clustal Omega (Multiple Sequence Alignment) version 1.2.1 (https://www.ebi.ac.uk/Tools/msa/clustalo/). In every case, both the nucleotide and the amino acid sequences were compared with the PAO1 reference strain (GenBank accession no. AE004091.2).

## Results

### Prevalence and real-time PCR confirmation of *Pseudomonas aeruginosa* isolates

A total of 8,351 water samples were collected from 5 different locations (n = 152) and the prevalence of *P*. *aeruginosa* is shown in Table 2. The identity of all *P*. *aeruginosa* isolated strains was confirmed by real-time PCR. On the whole, 53 samples (53/8351, 0.63%) were positive for *P*. *aeruginosa*, of which 10/207 (4.83%) were from swimming pools, 10/1,684 (0.59%) from healthcare facilities, 19/1,518 (1.25%) from accommodation facilities, 9/4,500 (0.2%) from municipal waterworks and 5/235 (2.13%) from residential buildings.

**Table 2:** Prevalence of *Pseudomonas aeruginosa* in water samples from different locations.

Location	N°	Tot. water samples	Positive samples	Prevalence (%)
**Swimming pools**	**41**	207	**10**	**4.83**
pool water		153	5	3.26
inlet water		54	5	9.2
**Healthcare facilities**	**7**	1684	**10**	**0.59**
**Receptive facilities**	**72**	1518	**19**	**1.25**
**Municipal waterworks**	**2**	4500	**9**	**0.2**
**Residential buildings**	**30**	235	**5**	**2.13**
Total	152	8351	53	0.63%

### Antibiotic susceptibility profile

The antibiotic test was performed to establish susceptibility profiles to sixteen antimicrobial molecules belonging to 6 different classes. The results, summarized in Table 3, show that 46 *P*. *aeruginosa* isolates (86.8%) were susceptible to 13 tested antibiotics, while 5 isolates (9.43%) were resistant to imipenem, one isolate (1.88%) was resistant to Ticarcillin + Clavulanate, whereas one multi-resistant isolate (1.88%) was resistant to both Piperacillin and Ticarcillin + Clavulanate.

**Table 3:** Results of susceptibility testing of *Pseudomonas aeruginosa* (n = 53 isolates) of each antibiotic used.

	Disk content (μg)	Zone diameter breakpoint (mm)		Susceptible^a^		Resistant^a^	
		S ≥	R <	N. (%)	Inhibition zone Ø (average ± SD)	N. (%)	Inhibition zone Ø (average ± SD)
**Penicillins**							
Piperacillin	30	18	18	52 (98.11)	24.6 ± 2.13	1 (1.88)	14 ± 1.00
Piperacillin+Tazobactam	30–6	18	18	53 (100)	25.8 ± 1.65	0	-
Ticarcillin + Clavulanate	75–10	18	18	51 (96.22)	25.8 ± 2.17	2 (3.77)	12.8 ± 2.54
Ticarcillin	75	18	18	53 (100)	24.8 ± 3.46	0	-
**Cephalosporins**							
Cefepime	30	19	19	53 (100)	28.0 ± 2.35	0	-
Ceftazidime	10	16	16	53 (100)	25.2 ± 2.71	0	-
**Carbapenems**							
Doripenem	10	25	22	53 (100)	35.2 ± 4.52	0	-
Imipenem	10	20	17	48 (90.56)	29.6 ± 2.02	5 (9.43)	13.2 ± 1.40
Meropenem	10	24	18	53 (100)	34.7 ± 6.15	0	-
**Monobactams**							
Aztreonam	30	50	16	53 (100)	29.5 ± 2.31	0	-
**Fluoroquinolones**							
Ciprofloxacin	5	25	22	53 (100)	36.6 ± 2.96	0	-
Levofloxacin	5	20	17	53 (100)	30.0 ± 2.49	0	-
**Aminoglycosides**							
Amikacin	30	18	15	53 (100)	23.3 ± 2.40	0	-
Gentamicin	10	15	15	53 (100)	18.6 ± 2.17	0	-
Tobramycin	10	16	16	53 (100)	24.7 ± 1.95	0	-
Netilmicin	10	12	12	53 (100)	15.2 ± 2.59	0	-

^**a**^Data of susceptible (S) or resistant (R) isolates according to EUCAST clinical breakpoint (www.eucast.org). Average of three experiments. Isolates with intermediate susceptibility are included into the “susceptible” category.

Intermediate susceptibility to aztreonam was found for all 53 *P*. *aeruginosa* isolates. Three isolates from swimming pools showed intermediate susceptibility to meropenem, and 1 isolate from a swimming pool showed intermediate susceptibility to doripenem.

### RAPD fingerprinting of *Pseudomonas aeruginosa* isolates

**Fig 1:**
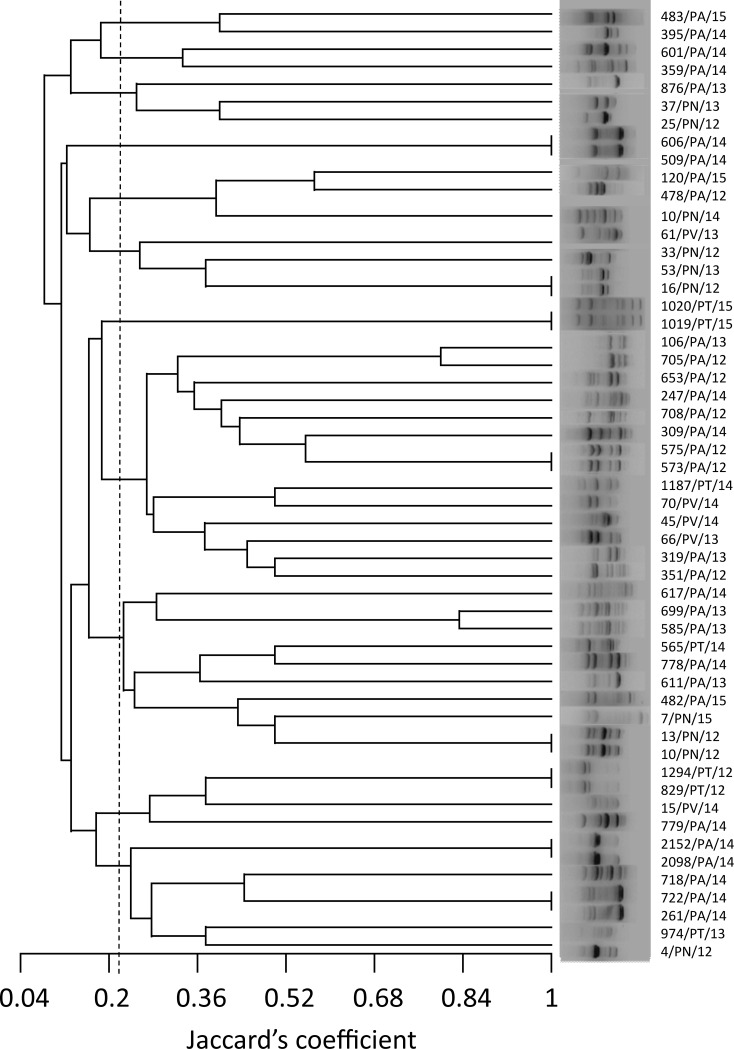
Dendrogram of random amplified polymorphic DNA (RAPD) analysis of 53 *P*. *aeruginosa* isolates. UPGMA clustering produced 12 groups at an arbitrary similarity level of 22% (Jaccard’s coefficient, dotted line). The methodology, using primer 272, revealed great diversity in genotypes (n = 45), identifying 37 unique genotypes and 8 clones.

The RAPD-derived banding pattern for the 53 *P*. *aeruginosa* isolates is shown in Fig 1.

Primer 272 produced clear banding patterns. The number of amplified DNA fragments for each strain ranged from 3 to 11, in a panel of 29 possible fragments of different molecular weight. RAPD banding patterns showed high diversity of genotypes (n = 45) among isolates, identifying 37 unique genotypes and 8 clones.

Cluster analysis was performed on the basis of similarity co-efficient generated from RAPD profiles. UPGMA clustering of RAPD data produced a dendrogram that could separate the 53 isolates into 12 groups at an arbitrary similarity level of 22% (Jaccard’s coefficient) (Fig 1).

### PCR detection of β-lactamases genes

Molecular screening tests carried out by the Xpert Carba R assay showed that Imipenem resistant *P*. *aeruginosa* isolates were β-lactamases-negative, at least for the main β-lactamase gene families.

### OprD sequence

The OprD amino acid sequences of the 11 isolated strains, deduced from the analysis of the *opr*D gene, were compared with the sequence of the PAO1 OprD. The OprD sequences were clustered into two major classes based on their susceptibility to imipenem (Table 4). The ‘susceptible strains’ major class, with 6 strains, was further classified into a total of 3 types of ‘full length type’ strains, whose OprD proteins were fully encoded. These 3 groups were characterized by different polymorphisms, which involved specific amino acid substitutions compared with the PAO1 OprD sequence. The second major group included the 5 imipenem resistant strains, which were clustered into three specific subgroups. Two strains, 10/PN/12 and 13/PN/12, showed several polymorphisms, which determined 4 amino acidic substitutions, and a frameshift mutation due to nucleotide deletion of 37 bp determining a variation of the amino acid sequence from the S349 and a premature stop codon after the 421 amino acid.

**Table 4:** Different OprD types and mechanisms of imipenem resistance detected among the *P*. *aeruginosa* isolates.

Imipenem resistance phenotype	Isolates	Alteration(s) of *opr*D nucleic sequence	Alteration(s) or amino acidic substitution(s) of OprD sequence^a^	Effect on OprD protein
**Susceptible strains**	4/PN/12, 25/PN/12, 33/PN/12, 37/PN/13	Several polymorphisms	D_43_N, S_57_E, **S**_**59**_**R**, **E**_**202**_**Q**, I_210_A, **E**_**230**_**K**, S_240_T, N_262_T, **A**_**267**_**S**, **A**_**281**_**G**, K_296_Q, Q_301_E, **R**_**310**_**G**, **V**_**359**_**L**, _372_(VDSSSS-YAGL)_383_	Full length type
	10/PN/14	Several polymorphisms	T_103_S, **K**_**115**_**T**, F_170_L	Full length type
	7/PN/15	Several polymorphisms	S_57_E, S_59_R, **V**_**127**_**L**, **E**_**185**_**Q**, **P**_**186**_**G**, **V**_**189**_**T**, E_202_Q, I_210_A, E_230_K, S_240_T, N_262_T, **T**_**276**_**A**, **A**_**281**_**G**, K_296_Q, Q_301_E, R_310_E, **A**_**315**_**G**, L_347_M, _372_(VDSSSS-YAGL)_383_, **S**_**403**_**A**, **Q**_**424**_**E**	Full length type
**Resistant strains**	10/PN/12, 13/PN/12	Several polymorphisms Deletion of 37 nt at position 1046 nt	D_43_N, T_103_S, **K**_**115**_**T**, F_170_L	Frameshift mutation, premature stop codon (shorter, nonfunctional protein)
			
	16/PN/12, 53/PN/13	Insertion of 1239 nt at position 215 nt		Premature stop codon and porin loss
			
	482/PA/15	Several polymorphisms Insertion of 1337 nt at position 428 nt	S_57_E, S_59_R, V_127_L	Premature stop codon and porin loss

^a^In bold, mutations which determine a charge change

+/-: K_115_T, V_127_L, V_189_T, A_267_S, V_359_L, Q_424_E

-/+: S_59_R, E_185_Q, E_202_Q, E_230_K, T_276_A, S_403_A

+/neutral: P_186_G, A_281_G, R_310_G, A_315_G

An insertion sequence (IS) element of 1239 bp, showing 99% identity with a transposase (CP008861.1, range from 3835714 to 3836949) was found in 16/PN/12 and 53/PN/13 strains at the nt 215 from the transcription start site. The IS presence caused a premature termination of translation after 84 aa and the loss of the OprD porin. Moreover, four amino acidic substitutions were detected in both protein sequences. An IS of 1,337 bp (99% identity with the transposase annotated in CP008857, ranging from 505,750 to 507,078) and 3 amino acidic substitutions were detected in 482/PA/15. Also in this case, the IS, at nt 428 from the transcription start, caused a premature stop codon at the 151 aa position and protein loss.

## Discussion

The evaluation of the microbiological quality of water aims to protect the general population from illness caused by contact with or ingestion of water that may contain pathogens such as *P*. *aeruginosa*. This opportunistic pathogen can be present in municipal water supplies and in water circuits offering suitable conditions for growth, and it poses potential health risks to particular segments of the population such as immunocompromised individuals, pregnant women, young children /infants and the elderly.

The present study investigates the presence of *P*. *aeruginosa* in different types of water obtained from a range of different environments. The supplementary bacteriological parameter *P*. *aeruginosa* was quantified for each water sample in accordance with Italian regulations for potable water [14], application of EC directive 98/83.

A very low overall prevalence of 0.63% was found in more than 8,000 samples collected over a 4-year period in swimming pool water, healthcare and accommodation facilities, municipal waterworks and residential buildings in the Marches Region.

The highest isolation rate (4.83%) of *P*. *aeruginosa* was observed in swimming pool water. This finding could be explained by the fact that colonized or infected individuals may use swimming pools [19].

In this study 7 out of the 41 (17%) investigated swimming pools were contaminated with at least one *P*. *aeruginosa* strain, and 2 out of the 41 (4.9%) were contaminated with two or more strains, although isolated in different samplings.

RAPD fingerprinting was used in order to identify sequence diversity and epidemiological relationships among isolates. The dendrogram (Fig 1) allowed us to cluster all isolates in 12 groups without any clear correlation with their origins. However, strains with identical RAPD profiles were isolated from the same location in the same year or even in different years. This result shows that a water system can remain colonized by one strain for long periods and the contamination may be difficult to eradicate.

Moreover, it highlights the importance of continuous monitoring and good maintenance of swimming pools because sand filtration and inactivation system may be inadequate to prevent pathogen contamination.

To achieve adequate chemical and microbiological quality and prevent the occurrence of undesirable transformations during storage and distribution, drinking water treatment is frequently recommended. Chlorination is commonly used to disinfect water because it is easy to apply at a moderate cost [28, 29]. Some studies have reported that water chlorination selects for antibiotic-resistant bacteria (ARB) [30–32]; however, this finding remains controversial [33,34].

In the present investigation, of a total of 53 *P*. *aeruginosa* strains isolated from 5 different locations, 7 (13.2%) isolates exhibited resistance to one or more antibiotics. Most of the strains (5/7, 72%) were resistant to imipenem. These strains were identified as carbapenemase-negative *P*. *aeruginosa* isolates. Resistance to imipenem has been associated with inactivating mutations within the *opr*D gene [35,36]. In particular, mutations in *opr*D caused by nucleotide insertions or deletions in the OprD structural gene have been found to be the major mechanisms leading to inactivation of OprD with a concomitant loss of porins from the *P*. *aeruginosa* outer membranes and increases in carbapenem MICs [37].

A recent study examining molecular mechanisms associated with carbapenem resistance in carbapenemase-negative *P*. *aeruginosa* isolates [38] showed no mutations in the promoter region of the *opr*D gene, but several in the coding region. Hence, sequencing analysis of the *opr*D gene was conducted to determine the mechanisms leading to imipenem resistance in 5 carbapenemase-negative *P*. *aeruginosa* isolates. Indeed, porin OprD is the specific point of entrance for carbapenems in *P*. *aeruginosa* [39], and mutational inactivation of the related gene is the main mechanism of carbapenem resistance in the absence of acquired carbapenemases [40]. The *opr*D sequence of 6 other imipenem sensitive strains isolated from recreational water in the present investigation was also determined for comparison. The sequence analysis of the susceptible strains revealed several amino acid substitutions, which can be clustered into three groups. In particular, the group of 4 strains composed of isolates 4/PN/12, 25/PN/12, 33/PN/12 and 37/PN/13, and strain 10/PN/14, showed the same amino acidic substitution patterns as the T1-VI and T1-II OprD types previously described by Ocampo-Sosa et al. [35], respectively. The 7/PN/15 isolate had the same alterations as the T1-VII group, except for the presence of P_186_G and the absence of D_437_E substitution [35]. Although some of the amino acid substitutions led to charge changes, the function of OprD porins was not affected, confirming the results of a previous study [35]. Moreover, the amino acid substitutions of the OprD sequence, described for the susceptible strains, were found in neither external loop 2 or 3, which are fundamental for imipenem entrance, nor in the deletion sites previously analyzed and related to imipenem susceptibility [37]. While loop 2 includes the imipenem binding site, loop 3 is more likely to be involved in the imipenem passage channel within OprD [37].

The 16/PN/12, 53/PN/13, and 482/PA/15 resistant strains encoded incomplete OprD proteins due to the presence of a stop codon caused by IS in the *opr*D gene, leading to the loss of the OprD ability to function as a porin. The IS elements were transposases inserted in different locations within the *opr*D, compared to other investigations [41,42].

In both the 10/PN/12 and 13/PN/12 isolates the *opr*D sequences showed 100% homology, with a 37-nucleotide deletion detected at 1,046 nt from the beginning of the coding region. The frameshift mutation determined an aberrant sequence of 72 aa from amino acid 349 positioned in loop 8, which begins at amino acid 345 [37]. This alteration may result in an abnormal protein structure and the loss of OprD function. Moreover, it should be noted that strains 10/PN/12 and 13/PN/12, which were isolated from the same swimming pool (from pool water and inlet water, respectively), also showed identical RAPD banding patterns (Fig 1), strongly suggesting an epidemiological correlation.

## Conclusion

The possible presence of *P*. *aeruginosa* in water distribution systems and swimming pools deserves attention given their potential as reservoirs or carriers of resistance or as opportunistic pathogens.

This study has revealed the presence of *P*. *aeruginosa* in potable and recreational water, including resistant strains, especially in swimming pools. Moreover, the present work identifies specific *opr*D mutations which are probably responsible for imipenem resistance and underlines the role of porins as a contributing factor in carbapenem resistance in Gram-negative bacteria.

Although imipenem is still considered a front-line antibiotic against *P*. *aeruginosa*, the results of this investigation confirm the continuous spread of resistance against the drug. This trend may pose a serious threat, especially if we consider that swimming pools are frequented by people with heterogeneous health statuses, including those who are potentially susceptible to opportunistic infections.

## Supporting information

S1 Table Sampling locations, strain and facilities identification codes. (DOCX)
