# Supplementary material for: Prevalence and antibiotic resistance of Pseudomonas aeruginosa in water samples in central Italy and molecular characterization of oprD in imipenem resistant isolates
Source: PLoS One. 2017 Dec 6;12(12):e0189172. doi: 10.1371/journal.pone.0189172 (PMC5718518; doi:10.1371/journal.pone.0189172)
Supplement: S1 Table — (DOCX) [file pone.0189172.s001.docx]

**S1 Table.** Sampling locations, strain and facilities identification codes.

Swimming pools

| **Strain n.** | **Sampling point** | **Facility** |
| --- | --- | --- |
| 4/PN/12 | inlet water | A |
| 10/PN/12 | pool water | B |
| 13/PN/12 | inlet water | B |
| 16/PN/12 | inlet water | C |
| 25/PN/12 | inlet water | D |
| 33/PN/12 | pool water | E |
| 37/PN/13 | pool water | F |
| 53/PN/13 | inlet water | C |
| 10/PN/14 | pool water | B |
| 7/PN/15 | pool water | G |

Healthcare facilities

| **Strain n.** | **Source** | **Facility** |
| --- | --- | --- |
| 351/PA/12 | hospital – hot water | A |
| 653/PA/12 | hospital – hot water | L |
| 106/PA/13 | hospital – hot water | B |
| 319/PA/13 | hospital – hot water | M |
| 247/PA/14 | hospital – hot water | C |
| 261/PA/14 | residential care home – hot water | D |
| 309/PA/14 | hospital – hot water | E |
| 395/PA/14 | hospital – hot water | C |
| 482/PA/15 | hospital – hot water | A |
| 483/PA/15 | hospital – hot water | A |

Accomodation facilities

| **Strain n.** | **Source** | **Facility** |
| --- | --- | --- |
| 478/PA/12 | camping – hot water | A |
| 573/PA/12 | hotel – hot water | B |
| 575/PA/12 | hotel– hot water | B |
| 705/PA/12 | hotel– hot water | I |
| 708/PA/12 | hotel– hot water | I |
| 585/PA/13 | hotel– hot water | B |
| 611/PA/13 | hotel– hot water | F |
| 699/PA/13 | hotel– hot water | B |
| 876/PA/13 | hotel– hot water | G |
| 359/PA/14 | hotel– hot water | C |
| 617/PA/14 | hotel– hot water | E |
| 601/PA/14 | hotel– hot water | F |
| 509/PA/14 | residence – hot water | D |
| 606/PA/14 | residence – hot water | D |
| 718/PA/14 | hotel– hot water | C |
| 722/PA/14 | hotel– hot water | C |
| 565/PT/14 | hotel– hot water | L |
| 779/PA/14 | temporary guesthouse– cold water | H |
| 120/PA/15 | temporary guesthouse – hot water | H |

Residential buildings

| **Strain n.** | **Source** |
| --- | --- |
| 61/PV/13 | household - tap water |
| 66/PV/13 | household - tap water |
| 45/PV/14 | household - tap water |
| 70/PV/14 | household - tap water |
| 778/PA/14 | household - tap water |

Municipal waterworks

| **Strain n.** | **Source** |
| --- | --- |
| 829/PT/12 | municipal water supply - outlet point |
| 1294/PT/12 | municipal water supply - outlet point |
| 974/PT/13 | municipal water supply - outlet point |
| 1187/PT/14 | municipal water supply - outlet point |
| 2098/PA/14 | municipal water supply - outlet point |
| 2152/PA/14 | municipal water supply - outlet point |
| 15/PV/14 | municipal water supply - outlet point |
| 1019/PT/15 | municipal water supply - outlet point |
| 1020/PT/15 | municipal water supply - outlet point |
